# Neighborhood Effects on Tree Mortality Depend on Life Stage of Neighbors

**DOI:** 10.3389/fpls.2022.838046

**Published:** 2022-02-22

**Authors:** Yi He, Heming Liu, Qingsong Yang, Ye Cao, Huimin Yin, Zhengkang Zhou, Qiuwu Yu, Xihua Wang

**Affiliations:** Zhejiang Tiantong Forest Ecosystem National Observation and Research Station, School of Ecological and Environmental Sciences, East China Normal University, Shanghai, China

**Keywords:** sapling, juvenile, adult, conspecific, heterospecific, species coexistence

## Abstract

Neighborhood effects are a crucial ecological processes that allow species to coexist in a forest. Conspecific and heterospecific neighbors, as major group classifications, affect tree mortality through various mechanisms associated with neighbor life stages. However, the influence of neighbor life stages on neighborhood effects and by what mechanisms remains a knowledge gap. Here we censused the mortality of 82,202 trees belonging to 30 species in a 20-ha subtropical forest and classified their neighbors into the following life stages: earlier, same and later. Next, we implemented generalized linear mixed models to estimate the effect of neighbors at different life stages on tree mortality. Our results showed that conspecific later stage neighbors had a positive effect on tree mortality overall, while conspecific earlier stage neighbors had a negative effect on tree mortality. Furthermore, these opposing effects appear to offset each other so that the overall effect of conspecific neighbors on tree mortality is weakened. In contrast, heterospecific neighbors had a decreasing effect on tree mortality overall. These effects are consistent with those of later stage heterospecific neighbors. Our findings demonstrate that neighbors strongly impact tree mortality, and their specific effects are closely related to neighbor life stages. Further, any single effect from one neighbor life stage may disturb or dominate the total effects of the neighbors. Therefore, the neighbors must be divided into different life stages to best explain the neighborhood effect on forest dynamics.

## Introduction

The neighbor effect has long been invoked to explain species coexistence and biodiversity maintenance in communities (Peters, 2003; Comita and Hubbell, 2009; Zhu et al., 2015; Fichtner et al., 2018; Glatthorn, 2021). Heterogeneous distributions of trees mainly arise due to the interactions between neighbors and focal trees (Silander and Pacala, 1985; Kim and Underwood, 2015; Pu et al., 2020). Resource enrichment and habitat amelioration can enhance positive interactions (Callaway and Walker, 1997; Brooker et al., 2008; Maestre et al., 2009; le Roux et al., 2013), while the spread of natural enemies and resource competition for limited resources can cause negative interactions (de Souza and Válio, 2001; Hyatt et al., 2003; Getzin et al., 2006; Gunton and Kunin, 2007; Fricke et al., 2014). These interactions regulate tree mortality patterns that maintain species coexistence (Zhang et al., 2009; Paine et al., 2012).

The conspecific neighbor effect is an important index of intraspecific interaction (Comita et al., 2014; Ding et al., 2019). Negative intraspecific interactions occur among aggregated conspecific individuals through resource competition and/or through encountering natural enemies (Janzen, 1970; Connell, 1971; Zhu et al., 2015; Liu et al., 2016, 2021). The strength of these negative intraspecific interactions increases with the density of conspecific neighbors. Trees tend to have a higher probability of mortality when conspecific neighbors are denser, closer, or more abundant (Castagneri et al., 2010; Comita et al., 2010; Johnson et al., 2012; Wang et al., 2012). This situation thereby provides space for heterospecific recruitment and promotes the coexistence of different species (Yao et al., 2020). However, positive intraspecific interactions may also occur among conspecifics due to similar habitat preferences and/or beneficial microorganisms (such as nitrogen-fixing bacteria) (Wu et al., 2016). Therefore, positive and negative interactions may exist simultaneously and act significantly as part of the conspecific neighbor effect.

The heterospecific neighbor effect is a synthesis of interspecific interactions. Previous studies have found that negative interspecific interactions typically result from strong heterospecific competition for resources (Tilman, 1987; Hubbell et al., 2001; Getzin et al., 2006). In addition, positive interspecific interactions are attributed to the herd protection hypothesis, which posits that heterospecific neighbors can effectively hinder the spread and reduce the risk of detection and of invasion of specific natural enemies such as pests or pathogens, thereby reducing individual mortality (Wills, 1996; Peters, 2003; Zhu et al., 2015; Yao et al., 2020). Further, if heterospecific neighbors exhibit similar habitat preferences as individuals, positive interspecific interactions may take place within those habitats (Lebrija-Trejos et al., 2014; Wu et al., 2016; Yao et al., 2020). Finally, some studies have found that the strength of negative and positive interspecific interactions likely correlates with the density of heterospecific neighbors (Peters, 2003; Wu et al., 2017).

Neighbors affect tree mortality *via* various mechanisms associating with their life stages. For example, conspecific neighbors of earlier life stages are typically offspring of focal adult trees. In this situation, the patterns of conspecific, earlier life stage neighbors around the focal adult tree are most likely due to dispersal limitation (Vincent et al., 2011). Here, the conspecific earlier life stage neighbors hardly affect the mortality of the parent tree (Weiner, 1990). In contrast, conspecific neighbors of the same or later life stages compete with the focal individual for resources and can further the spread of species-specific natural enemies, sometimes resulting in tree mortality (Weiner, 1990). Conspecific crowding may also be a result of shared habitat preference, and in that case, tree mortality decreases due to increased habitat suitability (Lebrija-Trejos et al., 2014; Wu et al., 2016; Yao et al., 2020). Similarly, the crowding of later-stage heterospecific neighbors leads to shading and fewer encounters between a host and its species-specific pests and pathogens. These heterospecific effects often reduce tree mortality (Wills, 1996; Peters, 2003).

Previous studies have shown that effects of conspecific and heterospecific neighbors influence tree mortality. However, the relative effects of neighbors of different life stages have yet to be described. To fill this research gap, we examined the effect of neighbors of different life stages on tree mortality in a subtropical forest through a generalized linear mixed modeling framework. Additionally, as environmental filtering is also an important mechanism contributing to tree mortality (Wang et al., 2012; Baldeck et al., 2013; Shen et al., 2014; Wu et al., 2017; Yao et al., 2020), environmental variables were considered in the models to account for environmental effects on tree mortality. Specifically, we addressed the following questions: (1) Do conspecific and heterospecific neighbors affect tree mortality? (2) If so, are there differences among the effects of neighbors at various life stages, both of the neighbors and of the focal trees, and how do these differences interact?

## Materials and Methods

### Study Site and Data Collection

**FIGURE 1:**
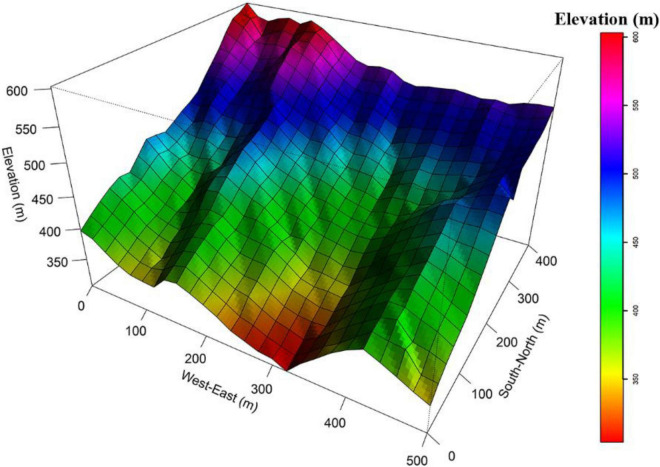
The topography of the 20-ha Tiantong forest dynamics plot.

This study was conducted in a 20-ha (500 m × 400 m) forest dynamics plot in a subtropical forest in the core zone of Tiantong National Forest Park (29° 48′ N, 121° 47′ E), (hereafter called the Tiantong plot), in Zhejiang province, eastern China. The forest community of this area is a mature evergreen broad-leaved forest because it neighbors Tiantong Temple and thus is protected as Fengshui forest without human disturbance for a long time. Here, the tree canopy is dominated by members of the Fagaceae and Theaceae families. The region is characterized by a typical monsoon climate with a hot, humid summers and a dry, cold winters. The minimum and maximum monthly mean temperatures are 4.2°C in January and 28.1°C in July, respectively. The mean annual temperature is 16.2°C. Average annual precipitation is 1,374.7 mm, which mainly occurs from May to August. The soil texture ranges from sandy to silty clay loam, with a pH values ranging from 4.4 to 5.1 (Song and Wang, 1995). The topography of the Tiantong plot is dynamic, with elevations ranging from 304.26 m to 602.89 m and slope ranging from 14° to 50° (Yang et al., 2011; Figure 1). All free-standing trees (DBH ≥ 1 cm) in the Tiantong plot were tagged, mapped, measured and identified to species in 2010 and re-censused in 2015 (following census standards for long-term large-scale forest dynamics plots) (Condit, 1998). In the 2010 census, the range of the DBH in Tiantong plot was 1–87.5 cm. The mean DBH of individuals in the plot was 5.66 cm. The diameter distribution of the forest followed a reversal J-shape. Individuals with DBH < 4 cm account for 64.15% of the total number of individuals in the forest (Yang et al., 2020). In the 2015 re-census, trees were defined as dead if the trunk was dead, broken, or if the stump was extracted.

### Life Stages and Focal Species

All species were classified into one of three life forms: shrub, subtree and canopy tree following the Flora of China (Wu and Peter, 2013). Each life form was divided into three life stages: sapling, juvenile and adult referring to their size-class structure (Yang et al., 2020) and according to their DBH (Peters, 2003; Baldeck et al., 2013; Piao et al., 2013; Zhu et al., 2015; Shi et al., 2018; Pu et al., 2020; Table 1). Samples of focal species were then selected of at least 50 individuals in each life stage, because, less than 50 individuals would cause data analyses inaccuracy. In total, 30 species met these requirements (species information in Supplementary Table 1). The total abundance of these focal species was 82,202 which accounted for 86.88% of all individuals in the Tiantong plot.

**TABLE 1:** DBH measurement criteria for dividing life stages of different life forms in the 20-ha Tiantong forest dynamics plot.

Life form	DBH range (cm)		
	Sapling	Juvenile	Adult
Shrub	<2 cm	2–4 cm	≥4 cm
Subtree	<3 cm	3–6 cm	≥6 cm
Canopy Tree	<5 cm	5–10 cm	≥10 cm

### Data Analyses

For the 2010 census, whole, alive trees of focal species were selected as samples. In the 2015 census, every sampled individual was classified as dead (1) or alive (0). The mortality of sapling, juvenile and adult individuals for all 20 m × 20 m grids were calculated, and the Wilcoxon signed-rank test was used to examine the differences in tree mortality across the life stages.

To examine the effects of neighbors of various life stages on tree mortality, we classified neighbors into three life stages: earlier life stage than the focal tree, same life stage as the focal tree and later life stage than the focal tree (see details in Table 2). Then, the neighborhood index was used to quantify the neighbor effect, which is defined as follows:


(1) $${\text{NI}}_{i}=\sum_{j}^{n}\left({\text{Ba}}_{j}/{\text{Distance}}_{j}\right)$$

**TABLE 2:** Division of different life stages of neighbors across ontogenetic stages of focal trees. All focal trees and neighbors were classified as saplings, juveniles or adults.

Ontogenetic stages	Life stage of neighbors		
	Earlier stage	Same stage	Later stage
Sapling stage		Sapling	Juvenile and Adult
Juvenile stage	Sapling	Juvenile	Adult
Adult stage	Sapling and Juvenile	Adult	

Where NI_*i*_ is the neighborhood index of focal tree *i*, *n* is the total number of neighbors, and *BA*_*j*_ is the basal area of neighbor *j*. Basal area is calculated by DBH [*Basalarea* = (DBH/2)^2^×π]. Here, Distance_*j*_ is the distance from neighbor *j* within the circle of optimal radius to focal tree *i*. The optimal radius is determined on model comparisons using different neighborhood radii (Zhu et al., 2015; Johnson et al., 2017; Liu et al., 2021). Generalized liner mixed models (GLMMs) with binomial errors were built to indicate the effects of conspecific and heterospecific neighbors on sapling, juvenile and adult mortality, respectively (where conspecific and heterospecific neighborhood index were treated as fixed variables, while grid and species were considered as the random variables). Neighbor radii were considered from 5 m to 40 m with 5 m step. Finally, an optimal radius was selected from the best model with the lowest *AIC* (Supplementary Table 2). The optimal radius of the neighbor effect differed among life forms.

The environmental variables were divided into topographic and soil variables. Topographic variables include factors such as elevation, slope, convexity and aspect. These variables were estimated within 5 m × 5 m quadrats. Altimetric points of the corners of 20 m × 20 m grids were first measured in the Tiantong plot, and then interpolated to the corners of the 5 m × 5 m quadrats using kriging. Elevation of each quadrat was defined as the mean value of its four corners (Harms et al., 2001; Valencia et al., 2004). Convexity was calculated as the quadrat’s elevation minus the mean elevation of the eight surrounding quadrats (Yamakura et al., 1995). By joining three corners of the quadrat to form a triangular plane, and each quadrat was divided into four triangular planes. Slope and aspect were determined as the deviation of the average angle of the four planes from the horizontal plane and the north direction, respectively (Lai et al., 2009). To ensure continuity of aspect data, a transformation of cos(α) + 1.1 was applied (Wang et al., 2007).

Total nitrogen (TN), total phosphorus (TP), pH value and soil moisture content were defined as soil variables. Soil samples were collected in 2011 following the 2010 census using the protocol defined by John et al. (2007) and Forest Global Earth Observatory^1^. In total, 1,310 sampling points were collected for the entire plot. Each soil sample was divided into two subsamples. One sub-sample of 10 mg was used to analyze total nitrogen content using an elemental analyzer (vario MICRO cube, Elementar, Germany). The other sub-sample of 350 mg was used to analyze total phosphorus content using a flow-injection auto-analyser (SAN++, Skalar, Netherlands). The unit of total nitrogen and phosphorus content was transformed into g/kg. Soil pH was determined using a Metterler Toledo pH meter (1:2, H_2_O). Soil moisture content was measured using the ring knife sampling method (volume of water in soil divided by volume of soil). These soil variables were also interpolated to the corners of the 5 m × 5 m quadrats using kriging.

Initial tree size can also affect tree mortality significantly, apart from neighborhood and environmental variables (Wang et al., 2012; Piao et al., 2013; Wu et al., 2017). Therefore, we also included a log-transformed measurement of size (DBH in 2010 census) as a potential variable. Additionally, to weaken the variation of baseline mortality among species and position, we defined species and grid number [each individual (DBH ≥ 1 cm) was assigned to a 20 m × 20 m grid] as random variables (Zhu et al., 2015).

GLMMs with binomial errors were used to examine the effects of potential variables on tree mortality. The GLMMs were specified as:


(2) $$\begin{array}{c} {\text{Y}}_{\mathrm{ijk}}∼\text{binomial}\,\left(1,{\text{p}}_{\mathrm{ijk}}\right)\\ \text{Ln}\,\left({\text{p}}_{\mathrm{ijk}}/\left(1-{\text{p}}_{\mathrm{ijk}}\right)\right)={\left[\alpha +\beta *\text{X}\right]}_{\mathrm{fixed}\,\mathrm{part}}\\ +{\left[\mu_{j}+\mu_{k}\right]}_{\mathrm{random}\,\mathrm{part}} \end{array}$$

Here ***Y_ijk_*** is 1 if individual ***i*** of species ***j*** in grid ***k*** was dead in the 2015 re-census, and 0 if otherwise, with ***P_ijk_*** as the predicted probability of being dead. Position (**μ*_k_***) and species (**μ*_j_***) are two random variables. **α** and **β** refer to an intercept and a vector of coefficients of explanatory variable ***X***, respectively.

*AIC* weight and model-average estimators of each potential explanatory variable were used to estimate their relative importance and effect, respectively. Methods of calculation were as follows: we first selected the m (from 0 to n) explanatory variables from the n potential explanatory variables (without repeated sampling), resulting in 2^n^ ($\sum_{i=0}^{n}C_{n}^{i}=2^{n}$) potential combinations. Then each combination was set as the fixed part of a GLMM, resulting in 2^n^ different GLMMs. Second, we calculated the *AIC* weight of each GLMM (Supplementary Equations 1, 2) and estimated the *AIC* weight of each potential explanatory variable (Supplementary Equations 3, 4). Finally, the optimal model group was selected (Δ*AIC* ≤ 2) and the model-average estimator and standard error (Supplementary Equations 5–8) of each potential explanatory variable was calculated in these models (Burnham and Anderson, 2002). This process for model selection reduces the influence of multiple-collinearity among potential explanatory variables and accounts for all influencing factors comprehensively (Liu et al., 2021).

To avoid edge effects (focal trees near edge of Tiantong plot are unable to select the whole neighbors), focal trees that were within 25 m of the edge of the Tiantong plot were excluded. All environmental factors were conditioned to avoid edge effects. Each continuous explanatory variable was standardized (by subtracting the mean value of the variable and dividing by one standard deviation) before all analyses. All analyses were performed in the R 3.5.1 computing environment (R Development Core Team 2018), using the “lme4 1.1-19” package (Bates et al., 2013). All statistical analyses were considered significant at the level of *P* < 0.05.

## Results

### Tree Mortality in Different Life Stages

**FIGURE 2:**
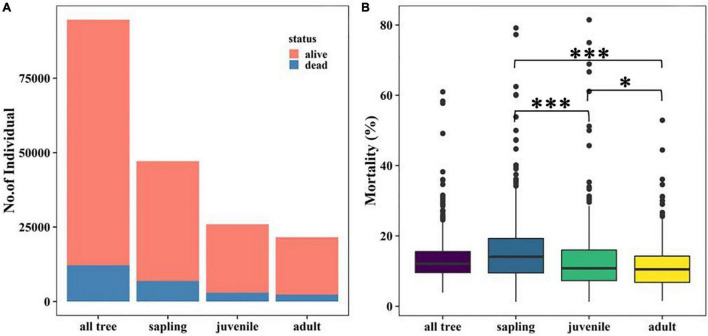
Tree abundance **(A)** and mortality **(B)** at different life stages. The significant effect of Wilcoxon signed-rank test is indicated by *P < 0.05, ^**^P < 0.01, and ^***^P < 0.001.

Tree mortality in the Tiantong plot was 12.88% overall. Saplings, the most abundant life form, suffered the highest mortality (14.63%), followed by juveniles and adults (Figure 2A). The median value of mortality in all grids also decreased significantly in the order of sapling > juvenile > adult (Figure 2B). Meanwhile, there were large variations of mortality among life forms and focal species (Supplementary Table 1 and Supplementary Figure 1).

### Conspecific and Heterospecific Neighbor Effects on Tree Mortality

**FIGURE 3:**
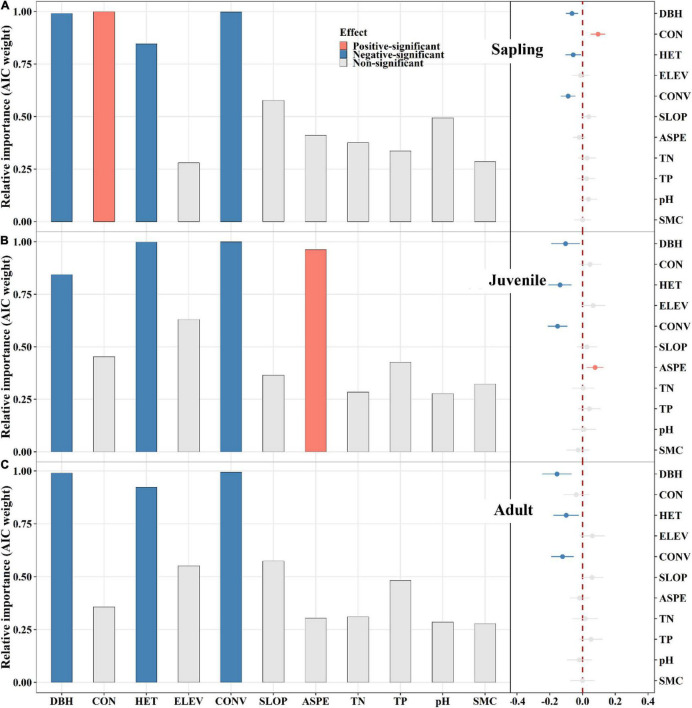
The relative influence of potential variables on tree mortality in sapling **(A)**, juvenile **(B)**, and adult **(C)** stage. The variables examined are the log-transformed initial DBH of tree (DBH), conspecific neighborhood index (CON), heterospecific neighborhood index (HET), elevation (ELEV), slope (SLOP), aspect (ASPE), convexity (CONV), total nitrogen (TN), total phosphorus (TP), pH value (pH) and moisture content (SMC) of soil. Error bars represent 1.96 $\overset{\wedge }{\bar{s.e._{j}}}$ around the model-average estimator ($\overset{\wedge }{\bar{\beta_{j}}}$). Blue and red points indicate that parameter estimates differ significantly from zero at the level of alpha = 0.05.

Conspecific neighbors had a significantly positive effect on sapling mortality (Figure 3A). In the juvenile and adult stages, conspecific neighbors did not exhibit significant effects (Figures 3B,C). Correspondingly, heterospecific neighbors displayed significantly negative effects on tree mortality in all stages (Figure 3). However, in the sapling stage, the conspecific neighbor effect was greater than that of heterospecific neighbors (Figure 3A). In addition, initial DBH and convexity exerted significantly negative effects on tree mortality across all stages (Figure 3), while aspect had a significantly positive effect on juvenile mortality (Figure 3B).

### Ontogenetic Neighbor Effects of Conspecifics and Heterospecifics on Tree Mortality

**FIGURE 4:**
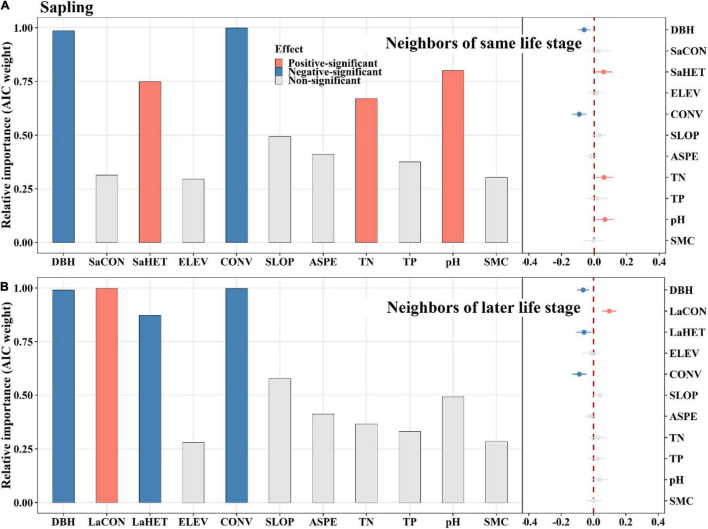
The relative influence of potential variables including neighbors of same life stage **(A)** or neighbors of later life stage **(B)** on sapling mortality. The variables examined are the log-transformed initial DBH of tree (DBH), conspecific neighborhood index at the same life stage (SaCON), conspecific neighborhood index at the later life stage (LaCON), heterospecific neighborhood index at the same life stage (SaHET), heterospecific neighborhood index at the later life stage indicates (LaHET), elevation (ELEV), slope (SLOP), aspect (ASPE), convexity (CONV), total nitrogen (TN), total phosphorus (TP), pH value (pH) and moisture content (SMC) of soil. Error bars represent 1.96 $\overset{\wedge }{\bar{s.e._{j}}}$ around the model-average estimator ($\overset{\wedge }{\bar{\beta_{j}}}$). Blue and red points indicate that parameter estimates differ significantly from zero at the level of alpha = 0.05.

**FIGURE 5:**
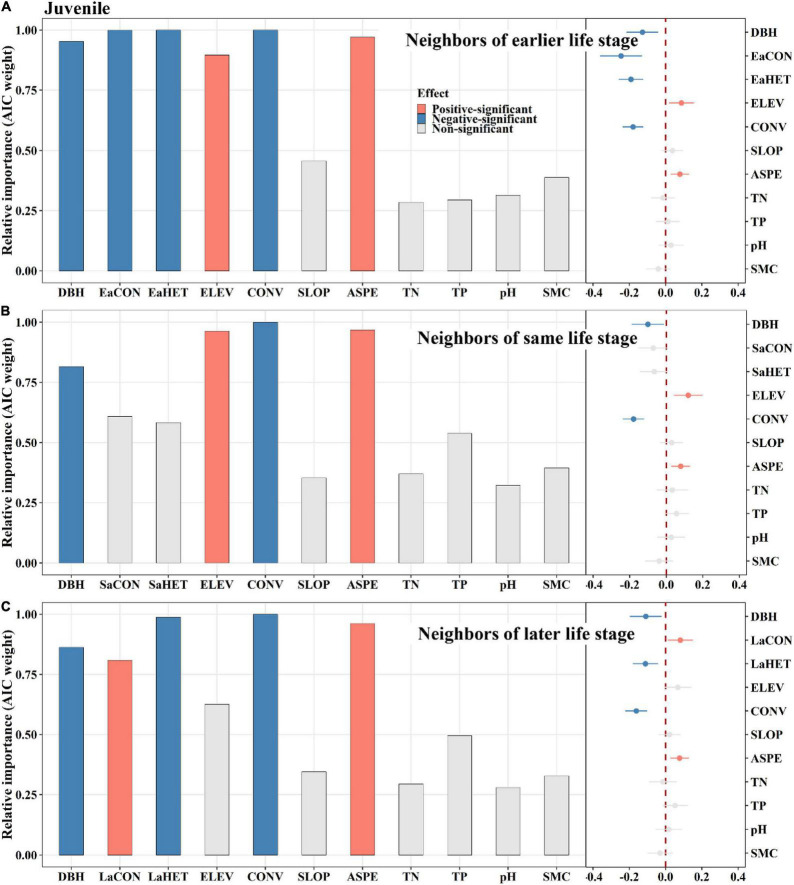
The relative influence of potential variables including neighbors of earlier life stage **(A)**, neighbors of same life stage **(B)** or neighbors of later life stage **(C)** on juvenile mortality. The variables examined are the log-transformed initial DBH of tree (DBH), conspecific neighborhood index at the earlier life stage (EaCON), conspecific neighborhood index at the same life stage (SaCON), conspecific neighborhood index at the later life stage (LaCON), heterospecific neighborhood index at the earlier life stage (EaHET), heterospecific neighborhood index at the same life stage (SaHET), heterospecific neighborhood index at the later life stage (LaCON), elevation (ELEV), slope (SLOP), aspect (ASPE), convexity (CONV), total nitrogen (TN), total phosphorus (TP), pH value (pH) and moisture content (SMC) of soil. Error bars represent 1.96 $\overset{\wedge }{\bar{s.e._{j}}}$ around the model-average estimator ($\overset{\wedge }{\bar{\beta_{j}}}$). Blue and red points indicate that parameter estimates differ significantly from zero at the level of alpha = 0.05.

**FIGURE 6:**
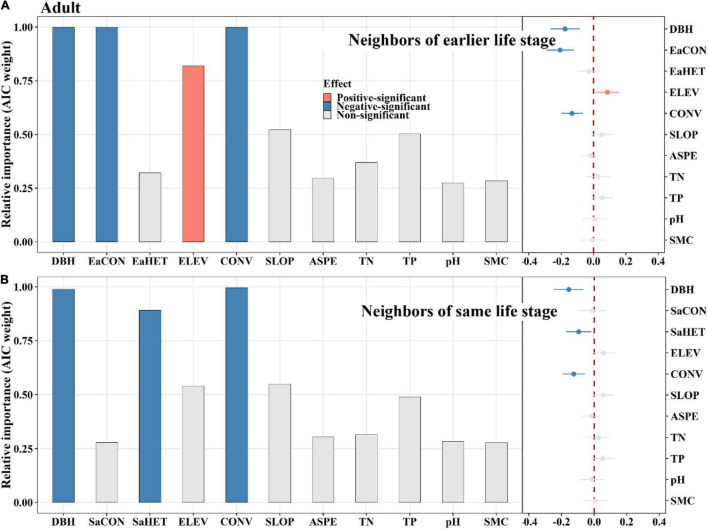
The relative influence of potential variables including neighbors of earlier life stage **(A)** or neighbors of same life stage **(B)** on adult mortality. The variables examined are the log-transformed initial DBH of tree (DBH), conspecific neighborhood index at the earlier life stage (EaCON), conspecific neighborhood index at the same life stage (SaCON), heterospecific neighborhood index at the earlier life stage (EaHET), heterospecific neighborhood index at the same life stage (SaHET), environmental variables including elevation (ELEV), slope (SLOP), aspect (ASPE), convexity (CONV), total nitrogen (TN), total phosphorus (TP), pH value (pH) and moisture content (SMC) of soil. Error bars represent 1.96 $\overset{\wedge }{\bar{s.e._{j}}}$ around the model-average estimator ($\overset{\wedge }{\bar{\beta_{j}}}$). Blue and red points indicate that parameter estimates differ significantly from zero at the level of alpha = 0.05.

Among saplings, conspecific later stage neighbors exerted a significantly positive effect on tree mortality, whereas same stage neighbors did not (Figure 4). Heterospecific same stage neighbors had a significantly positive effect on tree mortality, whereas later stage neighbors displayed an opposite effect (Figure 4). For juveniles, conspecific earlier stage neighbors had a significantly negative effect on tree mortality, whereas later stage neighbors had an opposite effect (Figures 5A,C). Heterospecific earlier and later neighbors exhibited significantly negative effects (Figures 5A,C). For adults, conspecific earlier stage and heterospecific same stage neighbors had significantly negative effects on tree mortality (Figure 6). In addition, TN and TP showed significantly positive effects on sapling mortality by offsetting the effect of later stage neighbors (Figure 4A). Elevation displayed significantly positive effects on juvenile and adult mortality by minimizing conspecific, later and same stage neighbor effects, respectively (Figures 5, 6).

## Discussion

Conspecific and heterospecific neighbors significantly affected ontogenetic tree mortality (Zhu et al., 2015). However, neighbors of different life stages different than that of the focal tree showed inconsistent effects (Koch et al., 2016). In our study, we found that conspecific earlier and later stage neighbors had opposite effects on juvenile mortality. Heterospecific same and later stage neighbors also had opposing effects on sapling mortality. The later stage neighbors were the only group whose singular effect was mostly consistent with the effect of all neighbors combined. In addition, failing to consider some life stages of neighbors may have resulted in the observation of related environmental factors showing more consequential effects.

Mortality attributed to conspecific negative density dependence is significantly widespread in the sapling stage, but this effect weakens as the tree grows (Zhu et al., 2015; Yao et al., 2020). Meanwhile, heterospecific neighbors reduce tree mortality throughout all life stages. Previous studies have shown that mortality due to conspecific negative density dependence is caused by specialized natural enemies and/or intraspecific competition for limited resources (Janzen, 1970; Connell, 1971; Zhu et al., 2015). Saplings, as the earliest life stage, suffer to a greater degree from natural enemy damage and from intraspecific competition than do older trees (Weiner, 1990). Therefore, saplings are more strongly impacted by conspecific neighbors. This result is consistent with findings of previous studies showing that density-dependent survival at the seedling and sapling stages plays a significant role in fostering tree species coexistence and maintaining diversity in forests (Bai et al., 2012; Johnson et al., 2014; Lin et al., 2014; Yan et al., 2015; Yao et al., 2020). Unlike conspecific neighbors, denser heterospecific neighbors can effectively hinder the spread of specific natural enemies and reduce objective tree mortality (Wills, 1996; Peters, 2003). This herd protection has also been observed in other studies of conspecific negative density dependence (CNDD) (Comita and Engelbrecht, 2009; Zhu et al., 2015; Yao et al., 2020). Furthermore, heterospecific neighbors displayed a negative correlation with adult tree mortality. This result indicates that heterospecific trees survive better around adult trees, which consequently promotes species coexistence.

Clumping of conspecific later stage and heterospecific same stage neighbors contributes the most to sapling mortality. Saplings are the youngest trees in this study, and as such are more susceptible to natural enemies and are less competitive. In this way, saplings may be subject to invasion upon the crowding of conspecific later stage neighbors because the neighbors could spread specific natural enemies, while also having asymmetric advantages in the competition for resources (Weiner, 1990; Clark and Clark, 1992; Uriarte et al., 2004; Yao et al., 2020). Consequently, conspecific later stage neighbors are the major contributor of all conspecific neighborhood effects to sapling mortality. In addition, conspecific later stage neighbors comprise the largest component of conspecific neighbors (Supplementary Figure 2). Thus, conspecific neighbors cause sapling mortality altogether (Figure 3A), although this effect can be largely attributed to conspecific later stage neighbors. This may also be why most studies of CNDD have found that the clumping of conspecific neighbors causes sapling mortality (Bai et al., 2012; Lin et al., 2014; Yan et al., 2015; Yao et al., 2020), even though these studies did not distinguish among conspecific neighbors’ life stages. In contrast, clumping of heterospecific later stage neighbors may reduce sapling mortality by hindering the spread of specific natural enemies (Peters, 2003). However, heterospecific sapling neighbors demonstrate the opposite effect, increasing focal sapling mortality. For one, herd protection is weakened in same stage heterospecific neighbors (Ramage et al., 2017), and they also have symmetric advantages in the competition for light resources, which can result in greater sapling mortality (Comita and Engelbrecht, 2009; Rüger et al., 2009; Bai et al., 2012; Piao et al., 2013).

Conspecific earlier and later stage neighbors show opposing effects on juvenile mortality, and this conflict destabilizes the overall conspecific neighbor effect. Conspecific earlier stage neighbors for juveniles are saplings, which are typically smaller and weaker than juveniles. Therefore, they hardly influence juvenile mortality (Weiner, 1990). However, the observation that conspecific sapling neighbors are clumped demonstrates that this habitat is suitable for regeneration (Grubb, 1977; Silvertown, 2004; Pérez-Ramos et al., 2012). The mortality of juveniles decreases correspondingly, and consequently, conspecific sapling neighbors significantly and negatively affect juvenile mortality. In contrast, adults are at a later stage than juveniles, and as mentioned above, they could also spread specific natural enemies and they have asymmetric competitive advantages compared to juveniles. However, juveniles are more adept at resisting natural enemies and asymmetric competition than are saplings (Boege and Marquis, 2005; DeMalach et al., 2016; Yao et al., 2020). Therefore, the positive effect of conspecific adult neighbors on juvenile mortality is weaker than it is on saplings. Furthermore, due to the weaker positive effect of conspecific adult neighbors and the counteraction of conspecific sapling neighbors, the overall conspecific neighbor effect on juvenile mortality is offset by these opposing effects. In our study, we were unable to even detect an overall conspecific neighbor effect on juvenile mortality (Figure 3B). Again, overlooking the life stages of neighbors when measuring their effect on focal tree mortality can result in a biased, incomplete conclusion.

Due to the positive spatial association between adult trees and their offspring, more living adult trees occur in the area with aggregations of earlier stage conspecific neighbors (Perea et al., 2021). Here, we found that when more conspecific earlier stage neighbors were clumped around the focal adult tree, its mortality was lower. Mature trees are generally surrounded by offspring due to dispersal limitation (Vincent et al., 2011). These dispersal patterns can have profound implications for later period, positive intraspecific spatial association (Moles et al., 2004; Beckman and Rogers, 2013; Sanín et al., 2013). Meanwhile, this positive intraspecific spatial association was observed to exhibit a greater degree around living adult trees than dead ones (Hou et al., 2004). Thus, clumping of conspecific earlier stage neighbors may reflect original dispersal limitation of focal adult trees with high survivability. In addition, although we considered the effects of environmental filtering on tree mortality by including several environmental factors in the analysis, other environmental factors that could have contributed to clumping of earlier stage conspecific neighbors and adult tree survival, such as canopy light, were excluded (Dechnik-Vázquez et al., 2016; Liu et al., 2017).

Environmental filtering is a vital process contributing to tree mortality across complex topographies (Wang et al., 2012; Yao et al., 2020; Andrus et al., 2021). The topography of the Tiantong plot is dynamic (Yang et al., 2011; Fang et al., 2017), with two parallel valleys in the north-south direction (Figure 1), and as such, convexity is considered to be an important environmental variable (Fang et al., 2017). In our study, trees tended to have a higher probability of mortality in low convexity habitats across all life stages. This may be because trees in lower convexity habitats may be disturbed more easily and experience less light availability. As light conditions are generally reduced from a south to north slope, juveniles occurring toward a north slope suffer higher mortality than juveniles near a south slope. In addition, initial tree size is consistently a strong predictor for tree survival (Wang et al., 2012; Ma et al., 2014; Wu et al., 2017). We also detected this effect across all life stages.

Environmental variables might have indirectly affected tree mortality through their correlations with the distributions of large neighbors. In this study, we classified the neighbors into different ontogenetic life stages. We found that removing some later stage neighbors from the analysis allowed the effects of some environment variables to become significant. These environmental variables were found to correlate with the variables of these later stage neighbors (Supplementary Figure 3). Therefore, this correlation supports the finding that the distribution of large individuals is influenced by environmental variables (Wang et al., 2012; Yao et al., 2020), meaning environmental variables likely affected tree mortality indirectly through neighborhood effects.

The findings of this study suggest that both conspecific and heterospecific neighbors play a major role in tree mortality (Peters, 2003; Zhu et al., 2015). However, these influences differ based on the life stage of the neighbors. Conspecific earlier stage neighbors generally have the effect of decreasing focal tree mortality because their clumping either reflects that the original dispersal limitation of the focal tree with high survivability or that the micro-habitat suitability for population colonization. In contrast, conspecific, later stage neighbors exert the effect of increasing focal tree mortality through CNDD. Furthermore, these opposing effects could minimize the overall conspecific neighbor effect. Due to herd protection, the assembling of heterospecific later stage neighbors could reduce focal tree mortality. In addition, large tree neighbors always dominate the neighborhood effects. At the same time, some environmental variables are closely correlated with the distribution of large trees. This correlation could result in the neighborhood effect on tree mortality, partially including an indirect environmental effect. Overall, these results suggest that there are multiple mechanisms among neighbors of different life stages in ontogenetic tree mortality, and thereby they highlight the necessity for dividing neighbors into different life stages when assessing the overall neighborhood effect contributing to species coexistence.

## Data Availability Statement

The datasets presented in this study can be found in online repository. The name of the repository and accession number can be found below: https://github.com/kkkliuheming/tree-mortality.

## Author Contributions

HL, YH, and XW conceived and designed the study. QSY, YC, HY, ZZ, and HL collected the data. YH and HL provided analysis tools and analyzed the data. HL, YH, QWY, QSY, and XW drafted and revised the article. All authors agreed to be accountable for all aspects of the work.

## Conflict of Interest

The authors declare that the research was conducted in the absence of any commercial or financial relationships that could be construed as a potential conflict of interest.

## Publisher’s Note

All claims expressed in this article are solely those of the authors and do not necessarily represent those of their affiliated organizations, or those of the publisher, the editors and the reviewers. Any product that may be evaluated in this article, or claim that may be made by its manufacturer, is not guaranteed or endorsed by the publisher.

## Footnotes

1
https://forestgeo.si.edu/sites/neotropics/barro-colorado-island/bci-maps/bci-soil-maps

## References

[B1] Andrus R. A. Chai R. K. Harvey B. J. Rodman K. C. Veblen T. T. (2021). Increasing rates of subalpine tree mortality linked to warmer and drier summers. *J. Ecol.* 109 2203–2218. 10.1111/1365-2745.13634

[B2] Bai X. Queenborough S. A. Wang X. Zhang J. Li B. Yuan Z. (2012). Effects of local biotic neighbors and habitat heterogeneity on tree and shrub seedling survival in an old-growth temperate forest. *Oecologia* 170 755–765. 10.1007/s00442-012-2348-2 22644047

[B3] Baldeck C. A. Harms K. E. Yavitt J. B. John R. Turner B. L. Valencia R. (2013). Habitat filtering across tree life stages in tropical forest communities. *Proc. R. Soc. B Biol. Sci.* 280:20130548. 10.1098/rspb.2013.0548 23843384 PMC3730581

[B4] Bates D. Maechler M. Bolker B. Walker S. (2013). *lme4: Linear Mixed-Effects Models Using Eigen and S4. R Package Version 3.2.1.*

[B5] Beckman N. G. Rogers H. S. (2013). Consequences of seed dispersal for plant recruitment in tropical forests: interactions within the seedscape. *Biotropica* 45 666–681. 10.1111/btp.12071

[B6] Boege K. Marquis R. J. (2005). Facing herbivory as you grow up: the ontogeny of resistance in plants. *Trends Ecol. Evol.* 20 441–448. 10.1016/j.tree.2005.05.001 16701415

[B7] Brooker R. W. Maestre F. T. Callaway R. M. Lortie C. L. Cavieres L. A. Kunstler G. (2008). Facilitation in plant communities: the past, the present, and the future. *J. Ecol.* 96 18–34.

[B8] Burnham K. P. Anderson D. R. (2002). *Model Selection and Multimodel Inference: A Practical Information-Theoretical Approach*, 2nd Edn. New York, NY: Springer.

[B9] Callaway R. M. Walker L. R. (1997). Competition and facilitation: a synthetic approach to interactions in plant communities. *Ecology* 78 1958–1965. 10.1016/j.biotechadv.2016.08.005 27587331

[B10] Castagneri D. Lingua E. Vacchiano G. Nola P. Motta R. (2010). Diachronic analysis of individual-tree mortality in a Norway spruce stand in the eastern Italian Alps. *Ann. For. Sci.* 67 304–304. 10.1051/forest/2009111

[B11] Clark D. A. Clark D. B. (1992). Life history diversity of canopy and emergent trees in a neotropical rain forest. *Ecol. Monogr.* 62 315–344. 10.2307/2937114

[B12] Comita L. S. Engelbrecht B. M. J. (2009). Seasonal and spatial variation in water availability drive habitat associations in a tropical forest. *Ecology* 90 2755–2765. 10.1890/08-1482.1 19886485

[B13] Comita L. S. Hubbell S. P. (2009). Local neighborhood and species’ shade tolerance influence survival in a diverse seedling bank. *Ecology* 90 328–334. 10.1890/08-0451.1 19323215

[B14] Comita L. S. Muller-Landau H. C. Aguilar S. Hubbell S. P. (2010). Asymmetric density dependence shapes species abundances in a tropical tree community. *Science* 329 330–332. 10.1126/science.1190772 20576853

[B15] Comita L. S. Queenborough S. A. Murphy S. J. Eck J. L. Xu K. Krishnadas M. (2014). Testing predictions of the Janzen-Connell hypothesis: a meta-analysis of experimental evidence for distance- and density-dependent seed and seedling survival. *J. Ecol.* 102 845–856. 10.1111/1365-2745.12232 25253908 PMC4140603

[B16] Condit R. (1998). *Tropical Forest Census Plots: Methods and Results From Barro Colorado Island, Panama and a Comparison With Other Plots.* Berlin: Springer. 10.1007/978-3-662-03664-8

[B17] Connell J. H. (1971). On the role of natural enemies in preventing competitive exclusion in some marine animals and in rain forest trees. *Dynamics of Populations* 298:312.

[B18] de Souza R. P. Válio I. F. M. (2001). Seed size, seed germination, and seedling survival of Brazilian tropical tree species differing in successional status. *Biotropica* 33 447–457. 10.1646/0006-3606(2001)033[0447:sssgas]2.0.co;2

[B19] Dechnik-Vázquez Y. A. Meave J. A. Pérez-García E. A. Gallardo-Cruz J. A. Romero-Romero M. A. (2016). The effect of treefall gaps on the understorey structure and composition of the tropical dry forest of Nizanda, Oaxaca, Mexico: implications for forest regeneration. *J. Trop. Ecol.* 32 89–106. 10.1017/s0266467416000092

[B20] DeMalach N. Zaady E. Weiner J. Kadmon R. (2016). Size asymmetry of resource competition and the structure of plant communities. *J. Ecol.* 104 899–910. 10.1371/journal.pone.0241913 33175854 PMC7657534

[B21] Ding Y. Zang R. Huang J. Xu Y. Lu X. Guo Z. (2019). Intraspecific trait variation and neighborhood competition drive community dynamics in an old-growth spruce forest in northwest China. *Sci. Total Environ.* 678 525–532. 10.1016/j.scitotenv.2019.05.014 31078842

[B22] Fang X. Shen G. Yang Q. Liu H. Ma Z. Deane D. C. (2017). Habitat heterogeneity explains mosaics of evergreen and deciduous trees at local-scales in a subtropical evergreen broad-leaved forest. *J. Veg. Sci.* 28 379–388. 10.1111/jvs.12496

[B23] Fichtner A. Härdtle W. Bruelheide H. Kunz M. Li Y. von Oheimb G. (2018). Neighbourhood interactions drive overyielding in mixed-species tree communities. *Nat. Commun.* 9:1144. 10.1038/s41467-018-03529-w 29559628 PMC5861250

[B24] Fricke E. C. Tewksbury J. J. Rogers H. S. (2014). Multiple natural enemies cause distance-dependent mortality at the seed-to-seedling transition. *Ecol. Lett.* 17 593–598. 10.1111/ele.12261 24589220

[B25] Getzin S. Dean C. He F. John A. Trofymow J. Wiegand K. (2006). Spatial patterns and competition of tree species in a douglas-fir chronosequence on Vancouver Island. *Ecography* 29 671–682. 10.1111/j.2006.0906-7590.04675.x

[B26] Glatthorn J. (2021). A spatially explicit index for tree species or trait diversity at neighborhood and stand level. *Ecol. Indic.* 130:108073. 10.1016/j.ecolind.2021.108073

[B27] Grubb P. J. (1977). The maintenance of species-richness in plant communities: the importance of the regeneration niche. *Biol. Rev.* 52 107–145. 10.1111/j.1469-185x.1977.tb01347.x

[B28] Gunton R. M. Kunin W. E. (2007). Density effects at multiple scales in an experimental plant population. *J. Ecol.* 95 435–445. 10.1111/j.1365-2745.2007.01226.x

[B29] Harms K. E. Condit R. Hubbell S. P. Foster R. B. (2001). Habitat associations of trees and shrubs in a 50-ha neotropical forest plot. *J. Ecol.* 89 947–959. 10.1111/j.1365-2745.2001.00615.x

[B30] Hou J. H. Mi X. C. Liu C. R. Ma K. P. (2004). Spatial patterns and associations in a Quercus-Betula forest in northern China. *J. Veg. Sci.* 15 407–414. 10.1111/j.1654-1103.2004.tb02278.x

[B31] Hubbell S. P. Ahumada J. A. Condit R. Foster R. B. (2001). Local neighborhood effects on long-term survival of individual trees in a neotropical forest. *Ecol. Res.* 16 859–875. 10.1046/j.1440-1703.2001.00445.x

[B32] Hyatt L. A. Rosenberg M. S. Howard T. G. Bole G. Fang W. Anastasia J. (2003). The distance dependence prediction of the Janzen-Connell hypothesis: a meta-analysis. *Oikos* 103 590–602. 25253908

[B33] Janzen D. H. (1970). Herbivores and the number of tree species in tropical forests. *Am. Nat.* 104 501–528. 10.1086/282687

[B34] John R. Dalling J. W. Harms K. E. Yavitt J. B. Stallard R. F. Mirabello M. (2007). Soil nutrients influence spatial distributions of tropical tree species. *Proc. Natl. Acad. Sci. U.S.A.* 104 864–869. 10.1073/pnas.0604666104 17215353 PMC1783405

[B35] Johnson D. J. Beaulieu W. T. Bever J. D. Clay K. (2012). Conspecific negative density dependence and forest diversity. *Science* 336 904–907. 10.1126/science.1220269 22605774

[B36] Johnson D. J. Bourg N. A. Howe R. McShea W. J. Wolf A. Clay K. (2014). Conspecific negative density-dependent mortality and the structure of temperate forests. *Ecology* 95 2493–2503. 10.1890/13-2098.1

[B37] Johnson D. J. Condit R. Hubbell S. P. Comita L. S. (2017). Abiotic niche partitioning and negative density dependence drive tree seedling survival in a tropical forest. *Proc. Biol. Sci.* 284:20172210. 10.1098/rspb.2017.2210 29237862 PMC5745420

[B38] Kim T. N. Underwood N. (2015). Plant neighborhood effects on herbivory: damage is both density and frequency dependent. *Ecology* 96 1431–1437. 10.1890/14-1097.1 26236855

[B39] Koch E. B. A. Camarota F. Vasconcelos H. L. (2016). Plant ontogeny as a conditionality factor in the protective effect of ants on a neotropical tree. *Biotropica* 48 198–205. 10.1111/btp.12264

[B40] Lai J. Mi X. Ren H. Ma K. (2009). Species-habitat associations change in a subtropical forest of China. *J. Veg. Sci.* 20 415–423. 10.1111/j.1654-1103.2009.01065.x

[B41] le Roux P. C. Shaw J. D. Chown S. L. (2013). Ontogenetic shifts in plant interactions vary with environmental severity and affect population structure. *New Phytol.* 200 241–250. 10.1111/nph.12349 23738758

[B42] Lebrija-Trejos E. Wright S. J. Hernández A. Reich P. B. (2014). Does relatedness matter? Phylogenetic density-dependent survival of seedlings in a tropical forest. *Ecology* 95 940–951. 10.1890/13-0623.1 24933813

[B43] Lin F. Comita L. S. Wang X. Bai X. Yuan Z. Xing D. (2014). The contribution of understory light availability and biotic neighborhood to seedling survival in secondary versus old-growth temperate forest. *Plant Ecol.* 215 795–807. 10.1007/s11258-014-0332-0

[B44] Liu H. Johnson D. J. Yang Q. Xu M. Ma Z. Fang X. (2021). The dynamics of conspecific tree and seedling neighbors on seedling survival in a subtropical forest. *For. Ecol. Manage.* 483:118924. 10.1016/j.foreco.2021.118924

[B45] Liu H. Ma Z. Yang Q. Fang X. Lin Q. Zong Y. (2017). Relationships between established seedling survival and growth in evergreen broad-leaved forest in Tiantong. *Biodivers. Sci.* 25 11–22. 10.17520/biods.2016290

[B46] Liu H. Shen G. Ma Z. Yang Q. Xia J. Fang X. (2016). Conspecific leaf litter-mediated effect of conspecific adult neighborhood on early-stage seedling survival in a subtropical forest. *Sci. Rep.* 6:37830. 10.1038/srep37830 27886275 PMC5122888

[B47] Ma L. Chen C. Shen Y. Wu L.-F. Huang Z.-L. Cao H.-L. (2014). Determinants of tree survival at local scale in a sub-tropical forest. *Ecol. Res.* 29 69–80. 10.1007/s11284-013-1100-7

[B48] Maestre F. T. Callaway R. M. Valladares F. Lortie C. J. (2009). Refining the stress-gradient hypothesis for competition and facilitation in plant communities. *J. Ecol.* 97 199–205. 10.1111/j.1365-2745.2008.01476.x

[B49] Moles A. T. Falster D. S. Leishman M. R. Westoby M. (2004). Small-seeded species produce more seeds per square metre of canopy per year, but not per individual per lifetime. *J. Ecol.* 92 384–396. 10.1111/j.0022-0477.2004.00880.x

[B50] Paine C. E. T. Norden N. Chave J. Forget P.-M. Fortunel C. Dexter K. G. (2012). Phylogenetic density dependence and environmental filtering predict seedling mortality in a tropical forest. *Ecol. Lett.* 15 34–41. 10.1111/j.1461-0248.2011.01705.x 22004454

[B51] Perea A. J. Wiegand T. Garrido J. L. Rey P. J. Alcántara J. M. (2021). Legacy effects of seed dispersal mechanisms shape the spatial interaction network of plant species in Mediterranean forests. *J. Ecol.* 109 3670–3684. 10.1111/1365-2745.13744

[B52] Pérez-Ramos I. M. Urbieta I. R. Zavala M. A. Marañón T. (2012). Ontogenetic conflicts and rank reversals in two Mediterranean oak species: implications for coexistence. *J. Ecol.* 100 467–477. 10.1111/j.1365-2745.2011.01912.x

[B53] Peters H. A. (2003). Neighbour-regulated mortality: the influence of positive and negative density dependence on tree populations in species-rich tropical forests. *Ecol. Lett.* 6 757–765. 10.1046/j.1461-0248.2003.00492.x

[B54] Piao T. Comita L. S. Jin G. Kim J. H. (2013). Density dependence across multiple life stages in a temperate old-growth forest of northeast China. *Oecologia* 172 207–217. 10.1007/s00442-012-2481-y 23053238 PMC3627022

[B55] Pu X. Umaña M. N. Jin G. (2020). Trait-mediated neighbor effects on plant survival depend on life stages and stage-specific traits in a temperate forest. *For. Ecol. Manage.* 472:118250. 10.1016/j.foreco.2020.118250

[B56] Ramage B. S. Johnson D. J. Gonzalez-Akre E. McShea W. J. Anderson-Teixeira K. J. Bourg N. A. (2017). Sapling growth rates reveal conspecific negative density dependence in a temperate forest. *Ecol. Evol.* 7 7661–7671. 10.1002/ece3.3298 29043023 PMC5632615

[B57] Rüger N. Huth A. Hubbell S. P. Condit R. (2009). Response of recruitment to light availability across a tropical lowland rain forest community. *J. Ecol.* 97 1360–1368. 10.1111/j.1365-2745.2009.01552.x

[B58] Sanín M. J. Anthelme F. Pintaud J.-C. Galeano G. Bernal R. (2013). Juvenile resilience and adult longevity explain residual populations of the Andean wax palm Ceroxylon quindiuense after deforestation. *PLoS One* 8:e74139. 10.1371/journal.pone.0074139 24194823 PMC3806763

[B59] Shen Y. Santiago L. S. Shen H. Ma L. Lian J. Cao H. (2014). Determinants of change in subtropical tree diameter growth with ontogenetic stage. *Oecologia* 175 1315–1324. 10.1007/s00442-014-2981-z 24938832

[B60] Shi W. Zhang Q. Sui X. Li B. He F. Chu C. (2018). The effects of habitat filtering and non-habitat processes on species spatial distribution vary across life stages. *Am. J. Bot.* 105 1469–1476. 10.1002/ajb2.1140 30098589

[B61] Silander J. A. Pacala S. W. (1985). Neighborhood predictors of plant performance. *Oecologia* 66 256–263. 10.1007/BF00379863 28311598

[B62] Silvertown J. (2004). Plant coexistence and the niche. *Trends Ecol. Evol.* 19 605–611. 10.1016/j.tree.2004.09.003

[B63] Song Y. Wang X. (1995). *Vegetation and Flora of Tiantong National Forest Park, Zhejiang Province.* Shanghai: Shanghai Scientific Documentary Press.

[B64] Tilman D. (1987). The importance of the mechanisms of interspecific competition. *Am. Nat.* 129 769–774. 10.1086/284672

[B65] Uriarte M. Canham C. D. Thompson J. Zimmerman J. K. (2004). A neighborhood analysis of tree growth and survival in a hurricane-driven tropical forest. *Ecol. Monogr.* 74 591–614. 10.1890/03-4031

[B66] Valencia R. Foster R. B. Villa G. Condit R. Svenning J.-C. Hernández C. (2004). Tree species distributions and local habitat variation in the Amazon: large forest plot in eastern Ecuador. *J. Ecol.* 92 214–229. 10.1111/j.0022-0477.2004.00876.x

[B67] Vincent G. Molino J.-F. Marescot L. Barkaoui K. Sabatier D. Freycon V. (2011). The relative importance of dispersal limitation and habitat preference in shaping spatial distribution of saplings in a tropical moist forest: a case study along a combination of hydromorphic and canopy disturbance gradients. *Ann. For. Sci.* 68 357–370. 10.1007/s13595-011-0024-z

[B68] Wang X. Comita L. S. Hao Z. Davies S. J. Ye J. Lin F. (2012). Local-scale drivers of tree survival in a temperate forest. *PLoS One* 7:e29469. 10.1371/journal.pone.0029469 22347996 PMC3278403

[B69] Wang X. Kent M. Fang X. (2007). Evergreen broad-leaved forest in Eastern China: its ecology and conservation and the importance of resprouting in forest restoration. *For. Ecol. Manage.* 245 76–87. 10.1016/j.foreco.2007.03.043

[B70] Weiner J. (1990). Asymmetric competition in plant populations. *Trends Ecol. Evol.* 5 360–364. 10.1016/0169-5347(90)90095-U 21232393

[B71] Wills C. (1996). Safety in diversity. *New Sci.* 149 38–42.

[B72] Wu H. Franklin S. B. Liu J. Lu Z. (2017). Relative importance of density dependence and topography on tree mortality in a subtropical mountain forest. *For. Ecol. Manage.* 384 169–179. 10.1016/j.foreco.2016.10.049

[B73] Wu J. Swenson N. G. Brown C. Zhang C. Yang J. Ci X. (2016). How does habitat filtering affect the detection of conspecific and phylogenetic density dependence? *Ecology* 97 1182–1193. 10.1890/14-2465.1 27349095

[B74] Wu Z. Peter H. R. (2013). *Flora of China.* Beijing: Science Press.

[B75] Yamakura T. Kanzaki M. Itoh A. Ohkubo T. Ogino K. Chai E. O. K. (1995). Topography of a large-scale research plot established within a tropical rain forest at Lambir, Sarawak. *Tropics* 5 41–56. 10.3759/tropics.5.41

[B76] Yan Y. Zhang C. Wang Y. Zhao X. von Gadow K. (2015). Drivers of seedling survival in a temperate forest and their relative importance at three stages of succession. *Ecol. Evol.* 5 4287–4299. 10.1002/ece3.1688 26664679 PMC4667830

[B77] Yang Q. Liu H. Yang H. Wang Z. Ma Z. Fang X. (2020). *Tiantong Subtropical Forest Dynamics Plot: Tree Species and Their Distribution Patterns.* Beijing: China Forestry Publishing House.

[B78] Yang Q. Ma Z. Xie Y. Zhang Z. Wang Z. Liu H. (2011). Community structure and species composition of an evergreen broad-leaved forest in Tiantong’s 20 ha dynamic plot, Zhejiang Province, eastern China. *Biodivers. Sci.* 19 215–223. 10.3724/SP.J.1003.2011.09013

[B79] Yao J. Bachelot B. Meng L. Qin J. Zhao X. Zhang C. (2020). Abiotic niche partitioning and negative density dependence across multiple life stages in a temperate forest in northeastern China. *J. Ecol.* 108 1299–1310. 10.1111/1365-2745.13335

[B80] Zhang J. Hao Z. Sun I. F. Song B. Ye J. Li B. (2009). Density dependence on tree survival in an old-growth temperate forest in northeastern China. *Ann. For. Sci.* 66 204–204. 10.1051/forest/2008086

[B81] Zhu Y. Comita L. S. Hubbell S. P. Ma K. (2015). Conspecific and phylogenetic density-dependent survival differs across life stages in a tropical forest. *J. Ecol.* 103 957–966. 10.1111/1365-2745.12414

