# Supplementary material for: Neighborhood Effects on Tree Mortality Depend on Life Stage of Neighbors
Source: Front Plant Sci. 2022 Feb 22;13:838046. doi: 10.3389/fpls.2022.838046 (PMC8902350; doi:10.3389/fpls.2022.838046)
Supplement: Supplementary file 1 [file Data_Sheet_1.PDF]

Supplementary material

Table S1. Abundance and mortality of different life stages in the 30 focal tree species (≥ 50 individuals in each life stage).

Species	Family	Leave properties	Life form	Abundance				Mortaliy		
				Sapling	Juvenile	Adult	All trees	Sapling	Juvenile	Adult
Eurya loquaiana	Theaceae	Evergreen	subtree	11489	4810	462	16761	4.85%	3.57%	8.70%
Litsea elongata	Lauraceae	Evergreen	subtree	4035	2232	2379	8646	21.09%	12.79%	9.13%
Camellia fraterna	Theaceae	Evergreen	shrub	3241	3281	805	7327	10.74%	8.25%	8.84%
Distylium myricoides	Hamamelidaceae	Evergreen	subtree	1982	791	1723	4496	14.87%	15.18%	11.43%
Neolitsea aurata var. *chekiangensis*	Lauraceae	Evergreen	subtree	1061	766	718	2545	13.01%	14.02%	15.68%
Symplocos anomala	Symplocaceae	Evergreen	subtree	1444	825	186	2455	23.92%	25.14%	33.88%
Machilus thunbergii	Lauraceae	Evergreen	canopy tree	1426	325	355	2106	22.03%	21.64%	7.66%
Cleyera japonica	Theaceae	Evergreen	subtree	844	337	822	2003	7.54%	5.33%	6.33%
Lithocarpus harlandii	Fagaceae	Evergreen	canopy tree	471	360	1024	1855	21.03%	12.71%	5.80%
Rhododendron ovatum	Ericaceae	Evergreen	subtree	808	702	297	1807	14.35%	11.32%	11.20%
Cyclobalanopsis sessilifolia	Fagaceae	Evergreen	canopy tree	530	427	779	1736	12.52%	8.56%	3.85%
Illicium lanceolatum	Illiciaceae	Evergreen	subtree	649	314	280	1243	15.73%	8.64%	9.48%
Symplocos sumuntia	Symplocaceae	Evergreen	subtree	800	307	74	1181	13.90%	16.76%	18.39%
Symplocos lucida	Symplocaceae	Evergreen	subtree	414	352	409	1175	23.10%	16.86%	13.08%
Machilus leptophylla	Lauraceae	Evergreen	canopy tree	577	87	397	1061	46.76%	29.52%	5.65%

Cinnamomum subavenium	Lauraceae	Evergreen	canopy tree	628	142	237	1007	20.93%	19.89%	6.47%
Schima superba	Theaceae	Evergreen	canopy tree	78	139	642	859	24.32%	12.50%	8.40%
Symplocos laurina	Symplocaceae	Evergreen	canopy tree	458	219	181	858	14.95%	7.09%	10.73%
Cyclobalanopsis myrsinaefolia	Fagaceae	Evergreen	canopy tree	262	423	144	829	29.92%	21.93%	12.50%
Carpinus viminea	Betulaceae	Deciduous	canopy tree	184	241	214	639	33.33%	8.82%	29.31%
Eurya muricata	Theaceae	Evergreen	shrub	225	278	98	601	6.79%	4.27%	6.98%
Photinia glabra	Rosaceae	Evergreen	subtree	160	251	154	565	17.95%	12.87%	7.14%
Ilex buergeri	Aquifoliaceae	Evergreen	canopy tree	295	86	159	540	6.40%	4.63%	15.35%
Alniphyllum fortunei	Styracaceae	Deciduous	canopy tree	198	152	166	516	37.87%	25.90%	13.51%
Daphniphyllum oldhami	Daphniphyllaceae	Evergreen	canopy tree	102	120	202	424	20.00%	10.29%	9.48%
Osmanthus cooperi	Oleaceae	Evergreen	canopy tree	210	96	102	408	4.53%	4.10%	3.45%
Syzygium buxifolium	Myrtaceae	Evergreen	shrub	122	158	107	387	9.21%	5.38%	3.25%
Styrax confusus	Styracaceae	Deciduous	subtree	153	124	82	359	20.93%	13.58%	7.02%
Symplocos lancifolia	Symplocaceae	Evergreen	subtree	98	84	127	309	13.68%	9.57%	9.09%
Lindera rubronervia	Lauraceae	Deciduous	subtree	132	84	60	276	32.53%	35.00%	36.84%

Table S2. *AIC* value of generalized linear mixed models with binomial errors to examine the effects of conspecific and heterospecific neighbors with different radii on tree mortality across life stages. The bold number highlights the lowest value of the differing neighbor radii in each life stage.

Radius	Sapling		Juvenile		Adult	
	AIC	Δ*AIC*	*AIC*	Δ*AIC*	*AIC*	Δ*AIC*
**5m**	21147.32	642.72	10425.21	196.63	7935.89	58.20
**10m**	21802.00	1297.40	10449.07	220.50	**7877.69**	**0.00**
**15m**	22567.90	2063.30	10849.87	621.30	8507.98	630.29
**20m**	22846.97	2342.37	11011.45	782.88	8775.87	898.18
**25m**	**20504.60**	**0.00**	**10228.57**	**0.00**	8594.09	716.40
**30m**	20602.45	97.85	10271.19	42.62	8635.96	758.27
**35m**	20660.67	156.07	10296.79	68.22	8664.17	786.48
**40m**	20713.89	209.29	10323.07	94.50	8692.27	814.58

Table S3. The optimal models for describing tree mortality at each life stage. The variables examined are the log-transformed initial DBH of tree (DBH), conspecific neighborhood index (CON), heterospecific neighborhood index (HET), elevation (ELEV), slope (SLOP), aspect (ASPE), convexity (CONV), total nitrogen (TN), total phosphorus (TP), pH value (pH) and moisture content (SMC) of soil.

Life stage	Model	*AIC*	Δ*AIC*
Sapling	DBH+CON+HET+SLOP+CONV	21306.3299	0.0000
	DBH+CON+HET+SLOP+CONV+pH	21306.7361	0.4062
	DBH+CON+HET+CONV	21306.8102	0.4803
	DBH+CON+HET+SLOP+CONV+ASPE	21307.0764	0.7465
	DBH+CON+HET+SLOP+CONV+pH+TN	21307.3912	1.0613
	DBH+CON+HET+SLOP+CONV+ASPE+pH	21307.4429	1.1131
	DBH+CON+HET+CONV+ASPE	21307.5223	1.1924
	DBH+CON+HET+CONV+pH	21307.5508	1.2209
	DBH+CON+HET+SLOP+CONV+TP	21307.6721	1.3423
	DBH+CON+HET+SLOP+CONV+pH+TP	21307.6994	1.3695
	DBH+CON+HET+SLOP+CONV+TN	21307.7806	1.4508
	DBH+CON+HET+SLOP+CONV+ASPE+pH+TN	21308.1127	1.7828
	DBH+CON+HET+CONV+pH+TN	21308.1167	1.7868
	DBH+CON+HET+ELEV+SLOP+CONV	21308.1230	1.7931
	DBH+CON+HET+CONV+TN	21308.1459	1.8160
	DBH+CON+HET+CONV+ASPE+pH	21308.2245	1.8947
	DBH+CON+HET+SLOP+CONV+SMC	21308.3294	1.9995
Juvenile	DBH+HET+ELEV+CONV+ASPE	10843.2028	0.0000
	DBH+CON+HET+ELEV+CONV+ASPE	10843.6034	0.4006
	DBH+HET+ELEV+CONV+ASPE+TP	10843.9104	0.7076
	DBH+CON+HET+ELEV+CONV+ASPE+TP	10844.0141	0.8113
	DBH+HET+CONV+ASPE	10844.1565	0.9537
	DBH+HET+ELEV+SLOP+CONV+ASPE	10844.5795	1.3766

	DBH+HET+ELEV+CONV+ASPE+SMC	10844.6348	1.4319
	DBH+CON+HET+CONV+ASPE	10844.6766	1.4738
	DBH+CON+HET+ELEV+CONV+ASPE+SMC	10844.9835	1.7807
	DBH+CON+HET+ELEV+SLOP+CONV+ASPE	10845.0356	1.8328
	DBH+HET+SLOP+CONV+ASPE	10845.0360	1.8332
	DBH+HET+CONV+ASPE+TP	10845.0569	1.8541
	DBH+HET+ELEV+CONV+ASPE+pH	10845.1491	1.9463
	DBH+HET+ELEV+SLOP+CONV+ASPE+TP	10845.1575	1.9547
	DBH+HET+ELEV+CONV+ASPE+TN	10845.1916	1.9888
Adult	DBH+HET+SLOP+CONV+TP	8053.1824	0.0000
	DBH+HET+ELEV+SLOP+CONV+TP	8053.1978	0.0154
	DBH+HET+ELEV+SLOP+CONV	8053.3407	0.1584
	DBH+HET+ELEV+CONV	8053.4484	0.2660
	DBH+HET+SLOP+CONV	8053.5756	0.3933
	DBH+HET+ELEV+CONV+TP	8053.6675	0.4852
	DBH+CON+HET+ELEV+SLOP+CONV+TP	8054.2630	1.0806
	DBH+CON+HET+ELEV+SLOP+CONV	8054.3058	1.1234
	DBH+CON+HET+SLOP+CONV+TP	8054.4021	1.2197
	DBH+CON+HET+ELEV+CONV	8054.5385	1.3561
	DBH+HET+CONV	8054.6526	1.4702
	DBH+HET+CONV+TP	8054.6699	1.4875
	DBH+HET+SLOP+CONV+TN	8054.6963	1.5139
	DBH+CON+HET+SLOP+CONV	8054.7023	1.5199
	DBH+HET+SLOP+CONV+ASPE+TP	8054.7836	1.6012
	DBH+CON+HET+ELEV+CONV+TP	8054.8514	1.6690

DBH+HET+ELEV+SLOP+CONV+ASPE+TP	8054.8607	1.6783
DBH+HET+ELEV+CONV+TN	8054.9763	1.7940
DBH+HET+SLOP+CONV+pH+TP	8055.0112	1.8288
DBH+HET+ELEV+SLOP+CONV+TN	8055.0177	1.8353
DBH+HET+ELEV+SLOP+CONV+ASPE	8055.0235	1.8411
DBH+HET+SLOP+CONV+TN+TP	8055.1275	1.9451
DBH+HET+ELEV+CONV+ASPE	8055.1374	1.9550
DBH+HET+SLOP+CONV+TP+SMC	8055.1449	1.9626
DBH+HET+ELEV+SLOP+CONV+TP+SMC	8055.1502	1.9678
DBH+HET+SLOP+CONV+pH	8055.1629	1.9805
DBH+HET+ELEV+SLOP+CONV+TN+TP	8055.1781	1.9957

Table S4. The optimal models for describing sapling mortality. The variables examined are the log-transformed initial DBH of tree (DBH), conspecific neighborhood index at the same stage (SaCON), conspecific neighborhood index at the later stage (LaCON), heterospecific neighborhood index at the same stage (SaHET), heterospecific neighborhood index at the later stage (LaHET), elevation (ELEV), slope (SLOP), aspect (ASPE), convexity (CONV), total nitrogen (TN), total phosphorus (TP), pH value (pH) and moisture content (SMC) of soil.

Ontogenetic stage relative to neighbors	Model	*AIC*	Δ*AIC*
Same life stage as neighbors	DBH+HET+CONV+pH+TN	21325.7343	0.0000
	DBH+HET+SLOP+CONV+pH+TN	21325.9768	0.2425
	DBH+HET+CONV+ASPE+pH+TN	21326.4164	0.6821
	DBH+HET+SLOP+CONV+ASPE+pH+TN	21326.6912	0.9569
	DBH+CON+HET+CONV+pH+TN	21327.2446	1.5103
	DBH+HET+CONV+pH+TN+TP	21327.3968	1.6625
	DBH+HET+SLOP+CONV+pH+TN+TP	21327.4367	1.7024
	DBH+HET+ELEV+CONV+pH+TN	21327.4436	1.7093
	DBH+CON+HET+SLOP+CONV+pH+TN	21327.5502	1.8158
	DBH+HET+CONV+pH+TN+SMC	21327.7218	1.9875
Later life stage than neighbors	DBH+CON+HET+SLOP+CONV	21304.8446	0.0000
	DBH+CON+HET+SLOP+CONV+pH	21305.2226	0.3780
	DBH+CON+HET+CONV	21305.3030	0.4584
	DBH+CON+HET+SLOP+CONV+ASPE	21305.5811	0.7365
	DBH+CON+HET+SLOP+CONV+ASPE+pH	21305.9192	1.0746
	DBH+CON+HET+SLOP+CONV+pH+TN	21305.9416	1.0970
	DBH+CON+HET+CONV+ASPE	21306.0049	1.1604
	DBH+CON+HET+CONV+pH	21306.0178	1.1733
	DBH+CON+HET+SLOP+CONV+TP	21306.2256	1.3810
	DBH+CON+HET+SLOP+CONV+pH+TP	21306.2302	1.3857
	DBH+CON+HET+SLOP+CONV+TN	21306.3383	1.4937
	DBH+CON+HET+ELEV+SLOP+CONV	21306.6149	1.7703

DBH+CON+HET+SLOP+CONV+ASPE+pH+TN	21306.6528	1.8082
DBH+CON+HET+CONV+pH+TN	21306.6567	1.8121
DBH+CON+HET+CONV+ASPE+pH	21306.6811	1.8365
DBH+CON+HET+CONV+TN	21306.6904	1.8458
DBH+CON+HET+SLOP+CONV+SMC	21306.8445	1.9999

Table S5 The optimal models for describing juvenile mortality. The variables examined are the log-transformed initial DBH of tree (DBH), conspecific neighborhood index at the earlier stage (EaCON), conspecific neighborhood index at the same stage (SaCON), conspecific neighborhood index at the later stage (LaCON), heterospecific neighborhood index at the earlier stage (EaHET), heterospecific neighborhood index at the same stage (SaHET), heterospecific neighborhood index at the later stage (LaHET), elevation (ELEV), slope (SLOP), aspect (ASPE), convexity (CONV), total nitrogen (TN), total phosphorus (TP), pH value (pH) and moisture content (SMC) of soil.

Ontogenetic stage relative to neighbors	Model	*AIC*	Δ*AIC*
Earlier life stage than neighbors	DBH+CON+HET+ELEV+CONV+ASPE	10815.2863	0.0000
	DBH+CON+HET+ELEV+SLOP+CONV+ASPE	10815.9794	0.6931
	DBH+CON+HET+ELEV+CONV+ASPE+SMC	10816.1094	0.8230
	DBH+CON+HET+ELEV+SLOP+CONV+ASPE+SMC	10816.3157	1.0293
	DBH+CON+HET+ELEV+CONV+ASPE+pH	10816.6720	1.3856
	DBH+CON+HET+ELEV+CONV+ASPE+TN	10817.0925	1.8062
	DBH+CON+HET+ELEV+CONV+ASPE+TP	10817.1379	1.8516
	DBH+CON+HET+ELEV+SLOP+CONV+ASPE+pH	10817.2288	1.9425
Same life stage as neighbors	DBH+CON+HET+ELEV+CONV+ASPE+TP	10855.4675	0.0000
	DBH+CON+HET+ELEV+CONV+ASPE	10855.6629	0.1954
	DBH+CON+ELEV+CONV+ASPE+TP	10856.0157	0.5482
	DBH+HET+ELEV+CONV+ASPE+TP	10856.1108	0.6433
	DBH+CON+HET+ELEV+SLOP+CONV+ASPE+TP	10856.5561	1.0886
	DBH+HET+ELEV+CONV+ASPE	10856.5947	1.1272
	DBH+CON+HET+ELEV+CONV+ASPE+TP+SMC	10856.6259	1.1584
	DBH+CON+HET+ELEV+CONV+ASPE+TN+SMC	10856.7748	1.3073
	DBH+ELEV+CONV+ASPE+TP	10856.8063	1.3388
	DBH+CON+HET+ELEV+SLOP+CONV+ASPE	10856.9078	1.4403
	DBH+CON+HET+ELEV+CONV+ASPE+SMC	10856.9141	1.4466
	DBH+CON+ELEV+CONV+ASPE	10856.9232	1.4558
	DBH+CON+ELEV+CONV+ASPE+pH+TP	10856.9741	1.5066
	DBH+CON+HET+ELEV+CONV+ASPE+pH+TP	10857.0353	1.5679

	DBH+CON+HET+ELEV+CONV+ASPE+TN	10857.1007	1.6332
	DBH+HET+ELEV+SLOP+CONV+ASPE+TP	10857.2898	1.8223
	DBH+HET+ELEV+CONV+ASPE+TP+SMC	10857.3058	1.8383
	DBH+CON+ELEV+CONV+ASPE+TP+SMC	10857.3183	1.8508
	DBH+CON+HET+ELEV+SLOP+CONV+ASPE+TP+SMC	10857.3645	1.8970
	DBH+CON+ELEV+SLOP+CONV+ASPE+TP	10857.4440	1.9765
	DBH+CON+HET+ELEV+CONV+ASPE+TN+TP	10857.4579	1.9904
	DBH+CON+HET+ELEV+CONV+ASPE+pH	10857.4646	1.9972
Later life stage than neighbors	DBH+CON+HET+ELEV+CONV+ASPE+TP	10843.5217	0.0000
	DBH+CON+HET+ELEV+CONV+ASPE	10843.6416	0.1199
	DBH+CON+HET+CONV+ASPE	10844.5491	1.0275
	DBH+CON+HET+CONV+ASPE+TP	10844.6885	1.1668
	DBH+CON+HET+ELEV+CONV+ASPE+TP+SMC	10844.7824	1.2607
	DBH+CON+HET+ELEV+SLOP+CONV+ASPE+TP	10844.9532	1.4316
	DBH+CON+HET+ELEV+CONV+ASPE+SMC	10845.0230	1.5014
	DBH+CON+HET+ELEV+SLOP+CONV+ASPE	10845.2184	1.6968
	DBH+CON+HET+ELEV+CONV+ASPE+pH+TP	10845.3236	1.8020
	DBH+CON+HET+ELEV+CONV+ASPE+TN+TP	10845.3767	1.8550

Table S6. The optimal models for describing adult mortality. The variables examined are the log-transformed initial DBH of tree (DBH), conspecific neighborhood index at the earlier stage (EaCON), conspecific neighborhood index at the same stage (SaCON), heterospecific

neighborhood index at the earlier stage (EaHET), heterospecific neighborhood index at the same stage (SaHET), elevation (ELEV), slope (SLOP), aspect (ASPE), convexity (CONV), total nitrogen (TN), total phosphorus (TP), pH value (pH) and moisture content (SMC) of soil.

Ontogenetic stage relative to neighbors	Model	*AIC*	Δ*AIC*
Earlier life stage than neighbors	DBH+CON+ELEV+SLOP+CONV+TP	8034.5294	0.0000
	DBH+CON+ELEV+CONV+TP	8034.6921	0.1627
	DBH+CON+ELEV+CONV	8034.9121	0.3827
	DBH+CON+ELEV+SLOP+CONV	8035.1309	0.6015
	DBH+CON+ELEV+CONV+TN	8035.6196	1.0902
	DBH+CON+HET+ELEV+SLOP+CONV+TP	8035.7970	1.2676
	DBH+CON+ELEV+SLOP+CONV+TN	8036.0735	1.5441
	DBH+CON+ELEV+SLOP+CONV+ASPE+TP	8036.2623	1.7329
	DBH+CON+HET+ELEV+CONV+TP	8036.2774	1.7479
	DBH+CON+HET+ELEV+SLOP+CONV	8036.3110	1.7816
	DBH+CON+HET+ELEV+CONV	8036.4109	1.8815
	DBH+CON+ELEV+CONV+ASPE+TP	8036.4300	1.9006
	DBH+CON+ELEV+SLOP+CONV+TN+TP	8036.4430	1.9136
	DBH+CON+ELEV+CONV+TN+TP	8036.4438	1.9144
	DBH+CON+ELEV+SLOP+CONV+pH+TP	8036.4859	1.9564
	DBH+CON+ELEV+SLOP+CONV+TP+SMC	8036.5007	1.9713
Same life stage as neighbors	DBH+HET+SLOP+CONV+TP	8053.7875	0.0000
	DBH+HET+ELEV+SLOP+CONV+TP	8053.9206	0.1331

DBH+HET+ELEV+CONV	8054.0774	0.2900
DBH+HET+ELEV+SLOP+CONV	8054.1246	0.3372
DBH+HET+SLOP+CONV	8054.2318	0.4443
DBH+HET+ELEV+CONV+TP	8054.2354	0.4479
DBH+HET+CONV+TP	8055.0439	1.2564
DBH+HET+CONV	8055.0769	1.2894
DBH+HET+SLOP+CONV+TN	8055.2851	1.4976
DBH+HET+SLOP+CONV+ASPE+TP	8055.3946	1.6071
DBH+HET+ELEV+CONV+TN	8055.5464	1.7589
DBH+HET+ELEV+SLOP+CONV+ASPE+TP	8055.5872	1.7997
DBH+HET+SLOP+CONV+pH+TP	8055.6557	1.8682
DBH+HET+CONV+TN	8055.7005	1.9130
DBH+CON+HET+SLOP+CONV+TP	8055.7135	1.9260
DBH+HET+SLOP+CONV+TN+TP	8055.7173	1.9298
DBH+HET+ELEV+SLOP+CONV+TN	8055.7412	1.9537
DBH+HET+SLOP+CONV+TP+SMC	8055.7440	1.9565
DBH+HET+ELEV+CONV+ASPE	8055.7694	1.9820

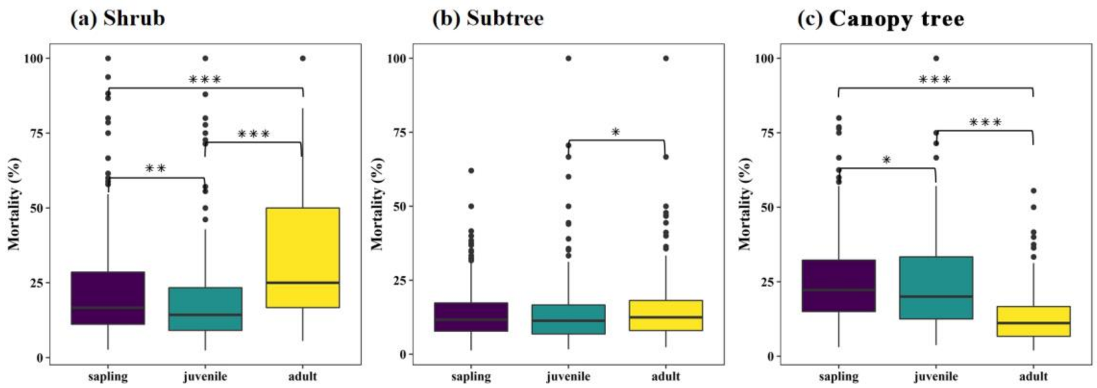

Figure S1 Tree mortality in three life forms (shrub, subtree and canopy tree) of different life stages. The significant effect of Wilcoxon signed-rank test is indicated by * (P<0.05), ** (P<0.01) and *** (P<0.001)

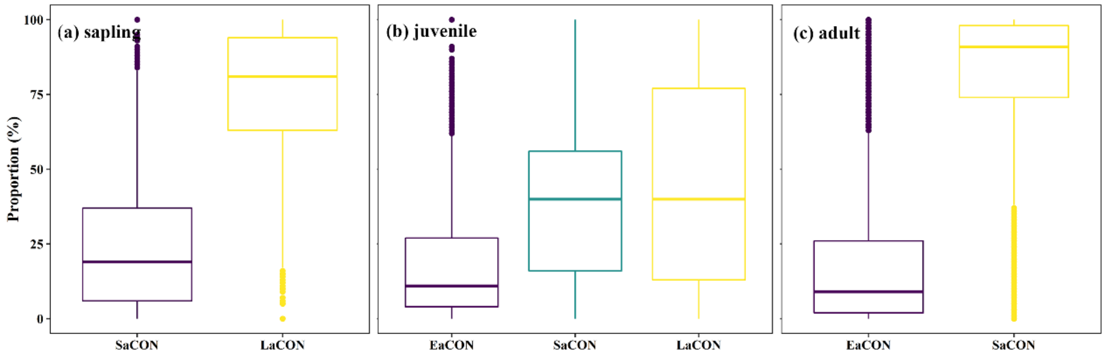

Figure S2. The proportion of conspecific neighbors in ontogenetic tree stages by life stage. EaCON indicates earlier life stage than focal tree; SaCON indicates same life stage; LaCON indicates later life stage.

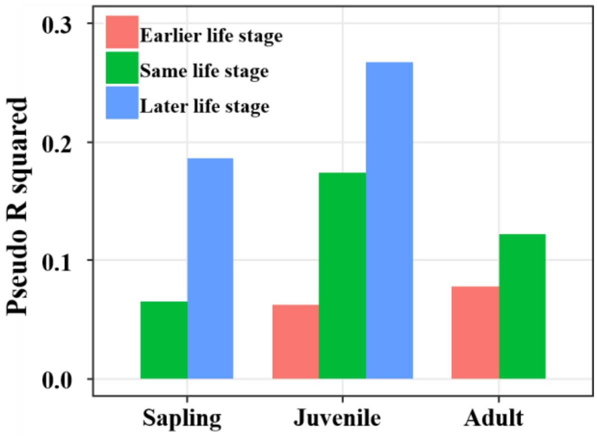

Figure S3. The marginal pseudo R-squared between the environmental variables and the tree life stages, by ontogenetic tree stage of neighbor.

Equations S1-S7

$$\Delta AIC_i = AIC_i - AIC_{min} \quad \text{(S1)}$$

$$w_i = \frac{\exp(-\frac{1}{2}\Delta AIC_i)}{\sum_{r=1}^{R}\exp(-\frac{1}{2}\Delta AIC_r)} \quad \text{(S2)}$$

$$I_j(g_i) = \begin{cases} 1 & \text{if potential explanatory variable } x_i \text{ is in model } g_i \\ 0 & \text{otherwise} \end{cases} \quad \text{(S3)}$$

$$w_+(j) = \sum_{i=1}^{R} w_i I_j(g_i) \quad \text{(S4)}$$

$$w_i' = \frac{\boldsymbol{exp}(-\frac{1}{2}\Delta AIC_i)}{\sum_{r=1}^{R1}\boldsymbol{exp}(-\frac{1}{2}\Delta AIC_r)} \quad \text{(S5)}$$

$$w_+'(j) = \sum_{i=1}^{R1} w_i' I_j(g_i) \quad \text{(S6)}$$

$$\overline{\hat{\beta}}_j = \frac{\sum_{i=1}^{R1} w_i' \beta_{j,i}}{w_+'(j)} \quad \text{(S7)}$$

$$\overline{\hat{s.e.}}_j = \frac{\sum_{i=1}^{R1} w_i' s.e._{j,i}}{w_+'(s.e.)} \quad \text{(S8)}$$

Here, AIC_i is the AIC of the focal model i and AIC_{min} is the minimum value of AIC in all R models. w_i represents AIC weight of the focal model i. $w_+(j)$ is AIC weight of potential explanatory variable j, which denotes the relative importance of this potential explanatory variable. w_i' and $w_+'(j)$ represent AIC weight of the focal optimal model i and potential explanatory variable j in the $R1$ optimal models. $\overline{\hat{\beta}}_j$ and $\overline{\hat{s.e.}}_j$ are the model-averaged estimator and the standard error of variable j in the optimal model groups, respectively.
